# miR-132 Regulates Dendritic Spine Structure by Direct Targeting of Matrix Metalloproteinase 9 mRNA

**DOI:** 10.1007/s12035-015-9383-z

**Published:** 2015-08-29

**Authors:** Magdalena Jasińska, Jacek Miłek, Iwona A. Cymerman, Szymon Łęski, Leszek Kaczmarek, Magdalena Dziembowska

**Affiliations:** 1 Laboratory of Neurobiology, The Nencki Institute, Pasteura 3, 02-093 Warsaw, Poland; 2 Laboratory of Molecular Basis of Synaptic Plasticity, Center of New Technologies, University of Warsaw, Warsaw, Poland; 3 School of Molecular Medicine, Żwirki i Wigury 61, 02-091 Warsaw, Poland; 4 Institute of Biochemistry and Biophysics, 02-106 Warsaw, Poland; 5 Laboratory of Molecular and Cellular Neurobiology, The International Institute of Molecular and Cell Biology, Warsaw, Poland; 6 Laboratory of Neuroinformatics, The Nencki Institute, Pasteura 3, 02-093 Warsaw, Poland

**Keywords:** Matrix metalloproteinase 9 (MMP-9), miR-132, Structural plasticity of dendritic spines

## Abstract

Mir-132 is a neuronal activity-regulated microRNA that controls the morphology of dendritic spines and neuronal transmission. Similar activities have recently been attributed to matrix metalloproteinase-9 (MMP-9), an extrasynaptic protease. In the present study, we provide evidence that miR-132 directly regulates MMP-9 mRNA in neurons to modulate synaptic plasticity. With the use of luciferase reporter system, we show that miR-132 binds to the 3’UTR of MMP-9 mRNA to regulate its expression in neurons. The overexpression of miR-132 in neurons reduces the level of endogenous MMP-9 protein secretion. In synaptoneurosomes, metabotropic glutamate receptor (mGluR)-induced signaling stimulates the dissociation of miR-132 from polyribosomal fractions and shifts it towards the messenger ribonucleoprotein (mRNP)-containing fraction. Furthermore, we demonstrate that the overexpression of miR-132 in the cultured hippocampal neurons from Fmr1 KO mice that have increased synaptic MMP-9 level provokes enlargement of the dendritic spine heads, a process previously implicated in enhanced synaptic plasticity. We propose that activity-dependent miR-132 regulates structural plasticity of dendritic spines through matrix metalloproteinase 9.

## Introduction

Reorganization of the neuronal networks supports physiological phenomena of learning and memory, as well as major neuropsychiatric pathologies, such as epilepsy, addiction, schizophrenia, to name just a few. The excitatory synapses located at the dendritic spines (small membranous protrusions extending from the dendrites) are the fundamental structural units of this circuitry that can be modulated in response to neuronal activity. Recently, microRNAs have emerged as important regulators of molecular events occurring at the synapses, and being responsible for their plastic changes.

MicroRNAs (miRNAs) are small, ~21-nt-long RNAs that post-transcriptionally regulate gene expression in eukaryotes. In animals, miRNAs bind to partially complementary sites in mRNAs, leading to translational repression and mRNA deadenylation and degradation [1–3]. Many studies confirmed the presence of microRNAs in the dendrites and synapses where they are believed to fine-tune the local expression of synaptic proteins [4]. Mir-132 is a neuronal activity-regulated microRNA, which expression is induced by plasticity-implicated transcription factor CREB—cAMP response element-binding protein [5]. MiR-132 was shown to be rapidly upregulated in model conditions of the synaptic plasticity, e.g., in the primary visual cortex after eye opening [6] and conversely, downregulated by mononuclear deprivation [7]. The expression levels of the primary and precursor forms of miR-212 and miR-132 were induced during long-term potentiation (LTP; an electrophysiological model of the synaptic plasticity) in the rat adult dentate gyrus [8] and upon brain-derived neurotrophic factor (BDNF) stimulation of primary cortical mouse neurons [9]. MiR-132 is present in the dendrites and at synapses [10–12], what makes it a potential unique regulator of locally translated synaptic proteins. Interestingly, the miR-132 knockout (resulting in miR-132/miR-212 double knockout, as they are coded by the same gene) is not essential for development or fertility of mice; however, its function is related to specific aspects of synaptic plasticity [13]. During the development of hippocampal neurons, miR-132 promotes dendritic arborization and neurite outgrowth [14–17]. In mature neurons, the effect of miR-132 is mostly related to the changes in spine morphology and synaptic transmission. [13]. When ectopically expressed in hippocampal neurons, miR-132 induces enlargement of dendritic spines [18, 6].

Matrix metalloproteinase-9 (MMP-9), an endopeptidase that regulates the pericellular environment through cleavage of its protein components, plays a critical role in the regulation of dendritic spine morphology and synaptic plasticity [19, 20]. Recently discovered local translation of MMP-9 mRNA at synapses in response to neuronal stimulation may provide major insight into mechanisms involving MMP-9 in the synaptic plasticity [21]. Furthermore, MMP-9 local translation was found to be regulated by fragile X mental retardation protein (FMRP). Mutations in its gene result in the most common form of inherited human mental retardation linked to autistic symptoms (fragile X syndrome, FXS), and respective knockout (Fmr1 KO) mice provide excellent animal model to approach the human condition. Recently, we demonstrated that in Fmr1 KO mice, the local, synaptic translation of MMP-9 mRNA is upregulated, what results in the higher MMP-9 activity at the synapse [22]. Locally secreted MMP-9 is involved in the reorganization of spine architecture [23, 24] and was shown to regulate spine morphology in Fmr1 KO mice [25]. One of the characteristic features of FXS neurons is the presence of immature, long, and thin dendritic spines [26, 27]. Interestingly, the genetic elimination of MMP-9 in Fmr1 KO mice by creating a double Fmr1/MMP-9 knockout mice rescued the aberrant phenotype of Fmr1 KO dendritic spines, ameliorated the abnormal mGluR5-dependent LTD, as well as aberrant behaviors [28]. Moreover, the reduction of exaggerated MMP9 mRNA translation in Fmr1(−/y) mice by the inhibition of translation initiation (reduction of eIF4E phosphorylation) also rescued core behavioral deficits of this mice as well as synaptic plasticity alterations and dendritic spine morphology defects [29]. These data show that the level of MMP-9 expression at the synapses is important for the maintenance of their proper structure and function.

Thus, both, miR-132 and MMP-9 have been implicated in the regulation of structural plasticity in neurons. However, no direct link between those important molecules has been revealed as yet. In the present study, we provide evidence for miR-132-dependent regulation of MMP-9 mRNA in neurons that result in structural changes of dendritic spines.

## Methods

### Animals

We have used early adult P26-P45 Fmr1 KO mice on FVB background as well as their wild-type littermates. Prior to the experiment, the animals were kept in the laboratory animal facility with free access to food and water with a 12 h light/dark cycle. All the procedures with animals were carried out according to guidelines of the First Warsaw Ethical Committee on animal research with appropriate permissions.

### Preparation of Synaptoneurosomes

Synaptoneurosomes were prepared from early adult P26-P45 wild-type and Fmr1 knockout mice by differential filtration as described previously [22]. Briefly, cortex and hippocampi from one mouse were dissected and homogenized on ice in 1 ml of homogenization buffer (containing (in mM) 125 NaCl, 1.2 MgSO4, 2.5 CaCl2, 1.53 KH2PO4, 212.7 glucose, 4 NaHCO3, pH 7.4 set with carbogen), supplemented with protease inhibitor cocktail (Sigma-Aldrich), and 100 U/ml mammalian placental RNase inhibitor (Fermentas). The final volume of the homogenate was set at 10 ml per one brain with homogenization buffer, and the samples were passed through a series of nylon mesh filters consecutively, 100, 60, 30, and 10 μm (Millipore Bedford, MA) and centrifuged at 1000×*g* for 15 min. The pellets containing synaptoneurosome preparations were washed once in the same volume of homogenization buffer, centrifuged as before, and resuspended in homogenization buffer to a final protein concentration of 1 mg/ml. Protein concentration was measured using BCA protein assay (Pierce).

### RNA Co-Immunoprecipitation and qRT-PCR

The immunoprecipitation with the anti-FMRP antibody (7G1 from Developmental Studies Hybridoma Bank) was performed as described previously [22]. Freshly prepared synaptoneurosomes were resuspended in 1200 μl of precipitation buffer (10 mM HEPES, pH 7.4, 400 mM NaCl, 30 mM EDTA, and 0.5 % Triton X-100) with protease inhibitor cocktail (Sigma-Aldrich) and 100 U/ml RiboLock (Fermentas). First samples were precleared with 120 μl of Dynabeads Protein A (Life Technologies) for 2.5 h. After preclearing, 100 μl of each supernatant was saved as an input fraction for Western blot to test procedure efficiency. Next, samples were precipitated overnight in 4 °C with 120 μl of antibody-bound Dynabeads Protein A, with either anti-FMRP antibody or normal mouse IgG. Total RNA was extracted with TRI-reagent (Sigma). For RNA isolated after FMRP-immunoprecipitation, the LSM mRNA (from *Arabidopsis thaliana*) was added into the reverse transcription reaction mix (6 pg per reaction) as the external control. Next, the RNA was reverse transcribed using random hexamer primers (Fermentas). Real-time PCR was performed using SYBRGreen PCR Master Mix (Applied Biosystems) and the following primers:

PSD95 (F:TGAGCTATGAGACGGTGACG, R:CGCTTAGGACGTGTCGTATG),

LSM (F: TCTTCTCTCTCCGTGTCCA, R:TGATCAATTCGCCAATGCG),

MMP-9 mRNA quantification after immunoprecipitation was performed using TaqMan PCR Master Mix (Applied Biosystems) and TaqMan primer/probe set for MMP-9 (Applied Biosystems, Mm00442991_m1). The quantification of miR-132 was carried out by using TaqMan MicroRNA Assay (Applied Biosystems). Values were calculated according to the ddCT method, using LSM mRNA as an external control.

### DNA Vectors

For the luciferase assays, the reporter constructs containing the coding sequence of firefly luciferase (FF-luc) and the untranslated 3’UTR sequence of rat MMP-9 mRNA under the synapsin 1 promoter (pSyn-Luc-3’UTR-MMP9) were prepared. pSyn-Luc-3’UTR-MMP9-MUT construct was obtained by site-directed mutagenesis of four nucleotides within the putative biding site for miR-132 in the pSyn-Luc-3’UTR-MMP9 plasmid.

The perfect seed match sequences for miR-132 or miR-9 were cloned downstream of the firefly luciferase in the FF-luc expressing vector. Constructs were prepared using the following primers:

miR132 seed match (L:CCCGACCATGGCTGTAGACTGTTAGGCGCGCCCGACCATG GCTGTAGACTGTTAGG, R:CCTAACAGTCTACAGCCATGGTCGGGCGCGCCTAACA GTCTACAGCCATGGTCGGG)

miR9 seed match (L:CCTCATACAGCTAGATAACCAAAGAGGCGCGCCTCATACA GCTAGATAACCAAAGAGG, R:CCTCTTTGGTTATCTAGCTGTATGAGGCGCGCCT CTTTGGTTATCTAGCTGTATGAGG)

MMP9-MUT (L:CGCTGTCCTTTCTTGTTGG**CAGT**TTTCTAATAAACACGGATCC, R:GGATCCGTGTTTATTAGAAA**ACTG**CCAACAAGAAAGGACAGCG).

The control vector expressing Renilla luciferase p-RL-CMV (Promega) was used for cotransfection and normalization of the results. pEZX-MR04 plasmid (GeneCopoeia) was used for the overexpression of miR-132.

### Primary Neuronal Cultures and Transfection

We have used different neuronal primary cell cultures for specific transfection protocols.

(i) For the efficient electroporation, freshly isolated and dissociated neurons from the P1 rat cortices were used. The optimized protocol allows for the 50 % of transfection efficiency. Cortical cultures prepared from P0 rat brains were used for luciferase assays. Cells were transfected after dissection and dissociation using Rat Neuron Nucleofector® Kit (Lonza) according to corresponding Amaxa™ Optimized Protocol. After transfection, neurons were cultured in Neurobasal-A medium (Life Technologies) supplemented with B27 (Life Technologies), glutamine, glutamate, and penicillin-streptomycin. Luciferase activity was analyzed 48–72 h after transfection.
(ii) For the transfection of differentiated rat cortical neurons at DIV7, we used NeuroMag Transfection Reagent (Ozbiosciences). We wanted to obtain sufficient transfection efficiency to perform the luciferase assay. The cells were transfected with pSyn-Luc-3’UTR-MMP9 reporter vector at DIV7 and incubated for additional 7 days. At 14 DIV, we quiescent spontaneous neuronal activity by inhibiting voltage-gated sodium channels (TTX), AMPA/kainate receptors (CNQX), NMDA receptors (APV), and L-type calcium channels (nifedipine—all from Sigma-Aldrich) for 10 h. After the lysis, the luciferase activity was analyzed in the silenced and untreated cells.
(iii) To asses the effect of miR-132 on the morphology of dendritic spines in the mature neurons, we have used primary hippocampal cultures (DIV14) obtained from Fmr1 KO and wt mice. The hippocampi were obtained from embryonic (E18) murine brains, dissociated and grown in neurobasal medium (Life Technologies) supplemented with B27 (Life Technologies), glutamine, glutamate, and penicillin-streptomycin. Neurons were transfected at DIV14 with vectors for miR-132 or EGFP overexpression using Lipofectamine® 2000 (Life Technologies). Cells were fixed 72 h after transfection (4 % paraformaldehyde/4 % sucrose in PBS) and immunostained with anti-GFP antibody (MAB3580 from Millipore).

### HEK293 Cell Culture and Transfection

HEK293 cells were cotransfected with reporter plasmids using polyethylenimine (PEI) (Sigma-Aldrich) and cultured in DMEM + glutamax medium (Life Technologies) supplemented with 10 % FBS and penicillin-streptomycin.

### Luciferase Assay

Luciferase activity was quantified 48 h after transfection using Dual-Glo® Luciferase Assay System (Promega) according to the manufacturer’s protocol. Relative expression of reporter constructs was determined by normalizing the ratio of FF-luc to RR-luc activity.

### Polyribosome Fractionation, Isolation of RNA, and Quantification

Synaptoneurosomes prepared from wt and Fmr1 KO mice were stimulated with 50 μM DHPG (Tocris Bioscience) for 15 min, as described previously [22]. Synaptoneurosomes were lysed using a buffer (20 mM Tris-HCl pH 7.4, 125 mM NaCl, 5 mM MgCl_2_, protease, and RNase inhibitors) containing 1.5 % Triton-X100, and membranous structures were removed by spinning at 20,000×*g* for 40 min. Resulting supernatant was loaded on a 10–50 % linear sucrose gradient (prepared in 20 mM Tris-HCl pH 7.5, 125 mM NaCl, and 5 mM MgCl_2_) and spun at 38,000 rpm for 2 h in SW41 rotor. Each gradient was separated into five fractions. Total RNA was isolated from each of the polysomal fractions using Tri-Reagent (Sigma) according to the manufacturer’s instruction. RNA was reverse transcribed using SuperScript III Reverse Transcriptase (Invitrogen) and random hexamer primers (Fermentas). Reverse transcription and qPCR of miR-132 was performed using TaqMan MicroRNA Assay (Applied Biosystems).

### Gel Zymography

Medium from cortical neurons transfected by electroporation with plasmids overexpressing miR-132 or EGFP was collected 36 h after transfection. The protein concentration was measured using the BSA kit (Pierce), and the equal concentration of the protein was mixed with 2× sample buffer Tris-Glycine SDS (Novex). Samples were subjected to electrophoresis under nondenaturing, nonreducing conditions in SDS-PAGE Tris-glycine 8 % acrylamide gels containing 0.5 % gelatin (POCH). Next, gels were washed twice for 20 min in 2.5 % Triton X-100 and incubated for 72 h in the zymography buffer (50 mM Tris, pH 7.5, 10 mM CaCl_2_, 1 μM ZnCl_2_, 1 % Triton X-100) at 37 °C. After incubation, gels were stained with 0.5 % Coomasie. The intensity of white bands on the blue background corresponding to the MMP-9 and matrix metalloproteinase 2 (MMP-2) activity was quantified with ImageJ program. The relative activity of MMP-9 was normalized to the MMP-2 activity that was constant in the analyzed samples.

### Western Blotting

Primary rat cortical neurons were transfected by electroporation with plasmids overexpressing miR-132 or EGFP. Cells were lysed 36 h after transfection in the 1 × SDS sample buffer, denatured and fractionated on 10 % SDS-polyacrylamide gels. The samples were electrotransferred onto PVDF membranes (Immobilon-P, Millipore), which were blocked 2 h at room temperature with 10 % nonfat milk in PBST. After blocking, the membranes were incubated at 4 °C overnight with anti-MMP-9 antibody (#3852, Cell Signaling) or anti-beta-actin (#A1978 Sigma) all diluted in 5 % nonfat milk PBST. Membranes were incubated 1 h at room temperature with peroxidase-labeled secondary antibody in 5 % nonfat milk in PBST and visualized with ECLplus reagent (GE Healthcare). The levels of MMP-9 and beta-actin were quantified using ImageJ software for three independent experiments. MMP-9 level was normalized to beta-actin.

To test the immunoprecipitation efficiency, Western blot with anti-FMRP antibody (#7104; Cell Signaling) was performed.

### Spine Clustering and Spine Parameters Estimation

Images of fixed, immunofluorescently stained neurons and dendritic spines were captured with LEICA TCS SP8 SMD confocal system. Spine area, length, and width were estimated using SpineMagick software [30]. Spine length was calculated as the length of the path from spine top to the dendrite along the virtual skeleton of a spine. For the purpose of shape analysis, the images of individual spines (obtained by semi-automatic segmentation) were first straightened, that is, transformed so that the virtual skeleton of each spine formed a straight line. Next, the images were rescaled to normalize the spine area, and for each spine we defined d(h), diameter of the spine as a function of distance from the dendrite. To classify the spines according to shape, we used a two-step procedure: first, all 6558 d(h) functions were clustered into 36 clusters; next, the clusters were manually sorted into three groups (mushroom, stubby, and thin spines) based on average images of the clusters and visual inspection of spines comprising the clusters.

To measure widths and lengths of necks and heads of mushroom spines, we approximated each normalized image with stereotyped shapes: a ball representing the spine head, a cylinder representing the spine neck, and a cone representing the part of a spine closest to the dendrite. Dimensions of fitted elements were then taken as dimensions of head and neck. Data analysis was performed using custom scripts written in Python, using NumPy and SciPy [31, 32] and Matplotlib [33].

## Results

### MMP-9 mRNA and miR-132 Associate with FMRP in Synaptoneurosomes

**Fig. 1:**
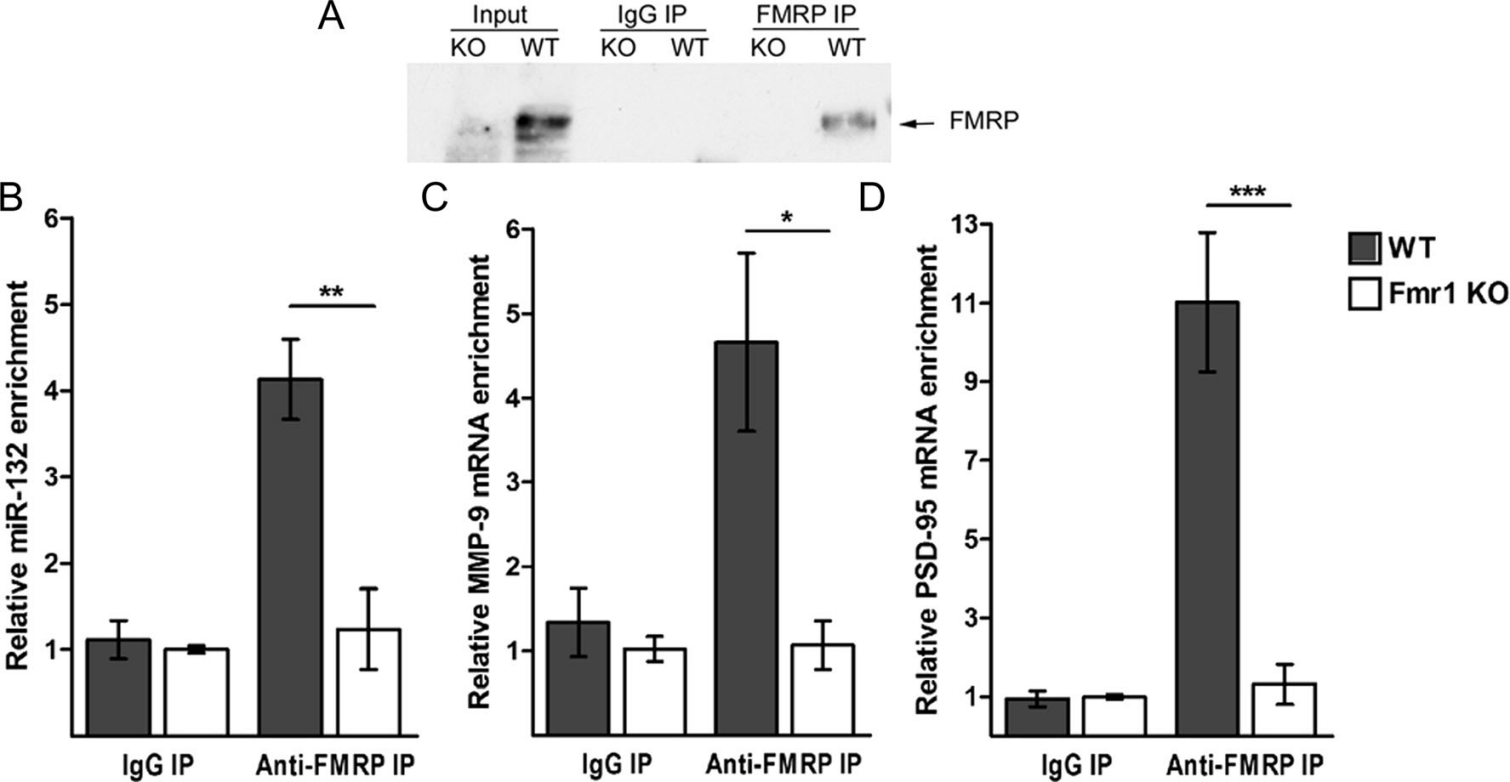
MMP-9 mRNA and miR-132 associate with FMRP in synaptoneurosomes. **a** MMP-9 and miR-132 recovery from FMRP-immunoprecipitation of wt and Fmr1 KO mice. Western blot analysis of the immunoprecipitated FMRP from mouse synaptoneurosomes shows FMRP precipitated by the anti-FMRP 7G1-1 antibody in wt mice. Fmr1 KO extracts as well as IgG IPs were used as negative controls. **b**–**d** RT-qPCR analysis of RNAs immunoprecipitated by anti-FMRP antibody—**b** miR-132, **c** MMP-9, and **d** PSD-95 (positive control) RNAs detected in the wt over Fmr1 KO immunoprecipitates. Values were normalized to the external “spike” control gene. *Error bars* indicate SEM, *n* = 3, *p < 0.03; **p < 0.01; ***p < 0.001 by Student’s *t* test

miR-132, as well as several other miRNAs, is associated with FMRP in mouse brain [18]. FMRP can also bind MMP-9 mRNA [22]. miR-132 and MMP-9 mRNA were not studied together in the context of FMRP; however, these findings suggested that they could be a part of the same protein-RNA complex. To answer this question, we have performed the RNA co-immunoprecipitation following the protocol used for the identification of MMP-9 mRNA as a target of FMRP with the anti-FMRP (7G1-1) antibody using protein extracts from synaptoneurosomes isolated from cerebral cortex and hippocampi of wt and Fmr1 KO mice. As shown in Fig. 1a, FMRP was precipitated with the 7G1-1 FMRP antibody in synaptoneurosomal extracts prepared from wild-type brains, while it was not present in the Fmr1 KO immunoprecipitates (IPs). To measure the amount of mature miR-132 and MMP-9 mRNA that coimmunoprecipitated with anti-FMRP antibody, we performed quantitative polymerase chain reaction (qPCR). As shown in Fig. 1b, c, both MMP-9 mRNA and miR-132 were significantly enriched in the wild-type FMRP precipitates when compared to the Fmr1 KO synaptoneurosomes, suggesting indirectly the interaction between those two RNAs. PSD-95 mRNA, a known target of FMRP [34], was also present in the FMRP complex (Fig. 1d). Moreover, we have checked the level of miR-132 expression in the hippocampi of wt and Fmr1 KO mice and we have not observed statistically significant differences in the level of miR-132 expression between the two genotypes, suggesting that basal miR-132 expression is not affected by the lack of FMRP protein.

### miR-132 Targets 3’UTR of MMP-9 mRNA and Regulates the Level of MMP-9 Protein in Neurons

**Fig. 2:**
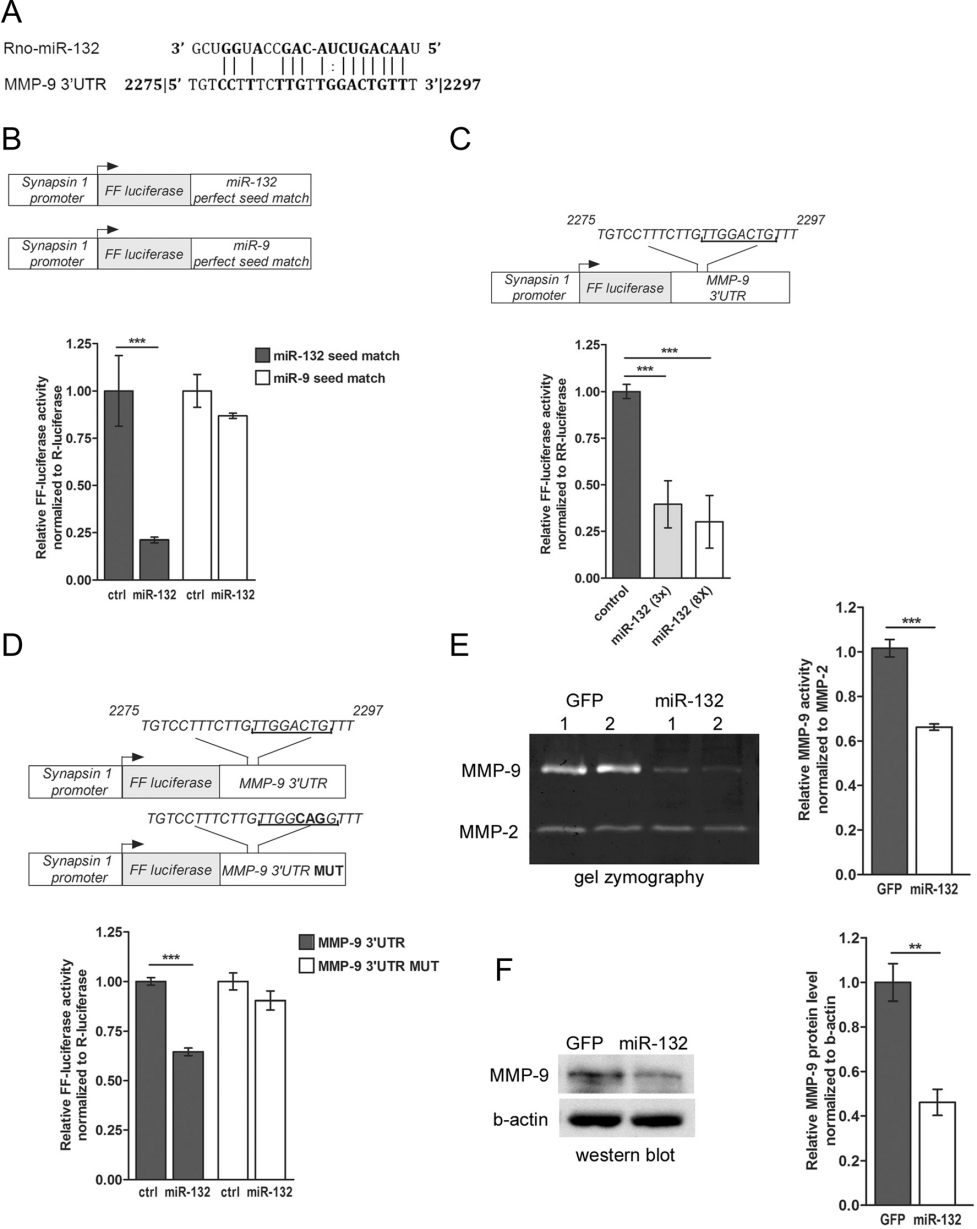
miR-132 targets 3’UTR of MMP-9 mRNA and regulates the level of MMP-9 activity in neurons. **a** Alignment of the murine 3’UTR sequence of MMP-9 with miR-132. **b** miRNA expression and activity in neurons was validated on FF-luc reporters containing perfect-match miRNA target-sites (sensors) *Error bars* indicate SEM, *n* = 5, ***p < 0.001 by Student’s *t* test. **c** Schematic representation of the luciferase reporter constructs—3′UTR of MMP-9 mRNA was fused to the coding sequence of firefly luciferase (*FF-luc*). The construct was validated in HEK cells. *Error bars* indicate SEM, *n* = 5, ***p < 0.001 by Student’s *t* test. **d** Schematic representation of the pSyn-Luc-3’UTR-MMP9 luciferase reporter construct and its mutated version—the mutation was introduced in the putative miR-132 target site of MMP-9 3’UTR, four nucleotides were changed. miR-132 failed to regulate the mutated MMP-9 3’UTR luciferase reporter, suggesting that it binds to the predicted sequence. The FF-luc reporter constructs were cotransfected with plasmid expressing miR-132 in primary rat cortical neurons, along with a plasmid encoding *Renilla reniformis* luciferase (*RR-luc*) for normalization. *Error bars* indicate SEM, *n* = 5, ***p < 0.001 by Student’s *t* test. **e** Cortical neurons were electroporated with EGFP or miR-132 precursor hairpin to overexpress the mature microRNA. The level of MMP-9 and MMP-2 was assessed in the culture medium from transfected cells by gel zymography. The activities of MMP-9 and MMP-2 were quantified by densitometry. *Error bars* indicate SEM, *n* = 5, ***p < 0.001 by Student’s *t* test. **f** Western blot analysis with specific anti-MMP-9 and anti-beta-actin antibodies. The level of MMP-9 was quantified and normalized to beta-actin. *Error bars* indicate SEM, *n* = 3, **p < 0.01 by Student’s *t* test

In silico analysis of the murine MMP-9 mRNA sequence revealed the presence of putative miR-132 binding site within the 3’UTR of the transcript (Fig. 2a). Furthermore, the binding region complementary to the miR-132 was found to be conserved between species. To determine whether miR-132 binds to the 3′ UTR of MMP-9 mRNA, we used the luciferase reporter assay. The coding sequence of firefly luciferase (FF-luc) was fused with the 3’UTR of MMP-9 mRNA, and, to ensure the efficient expression in neurons, luciferase was cloned under the control of synapsin 1 promoter. The expression of mature miR-132 from the commercial vector (pEZX-MR04 from Gene Copoeia) was validated on the FF-luc reporter constructs containing perfect-match miRNA target-sites for miR-132 and miR-9 (sensors). As shown in Fig. 2b, miR-132 specifically downregulates the expression of the miR-132 seed containing sensor but not the one containing the miR-9 sequence. The reporter construct was then cotransfected with the miR-132 expressing vector to the HEK293 cells together with a plasmid encoding *Renilla reniformis* luciferase (RR-luc), used for normalization. We have observed a dose-dependent effect of miR-132 inhibition on luciferase activity (Fig. 2c). To characterize the exact miR-132 binding site within the 3’UTR of MMP-9 transcript, the mutation was introduced into the putative miR-132 binding site (Fig. 2d). The cortical neurons in culture were transfected with the luciferase reporters together with the miR-132 overexpressing vector or EGFP expressing vector as a control. Overexpression of miR-132 in cortical neurons significantly reduced the luciferase activity by about 30 % compared to the control. Importantly, miR-132 failed to regulate the mutated MMP-9 3’UTR luciferase reporter, confirming the functionality of the predicted sequence within the 3’UTR of MMP-9 (Fig. 2d).

To validate if miR-132 can regulate the level of endogenous MMP-9 in neurons, we transfected primary cortical cultures with miR-132 precursor hairpin to overexpress the mature microRNA. MMP-9 is secreted by neurons in response to different types of synaptic stimulation; therefore, we measured its enzymatic activity in the cell culture medium using gel zymography. Overexpression of miR-132 reduced the level of secreted MMP-9 protein by about 40 % when compared to the control neurons transfected with EGFP expressing vector (Fig. 2e). In the same culture media samples, the level of matrix metalloproteinase 2 (MMP-2) did not change, indicating that mir-132 specifically regulates endogenous MMP-9 in neurons. Western blot analysis with specific anti-MMP-9 antibody further confirmed the downregulation of MMP-9 protein by miR-132 in neurons overexpressing miR-132 (Fig. 2f).

### Activity-Dependent Translation of MMP-9 Involves miR-132

**Fig. 3:**
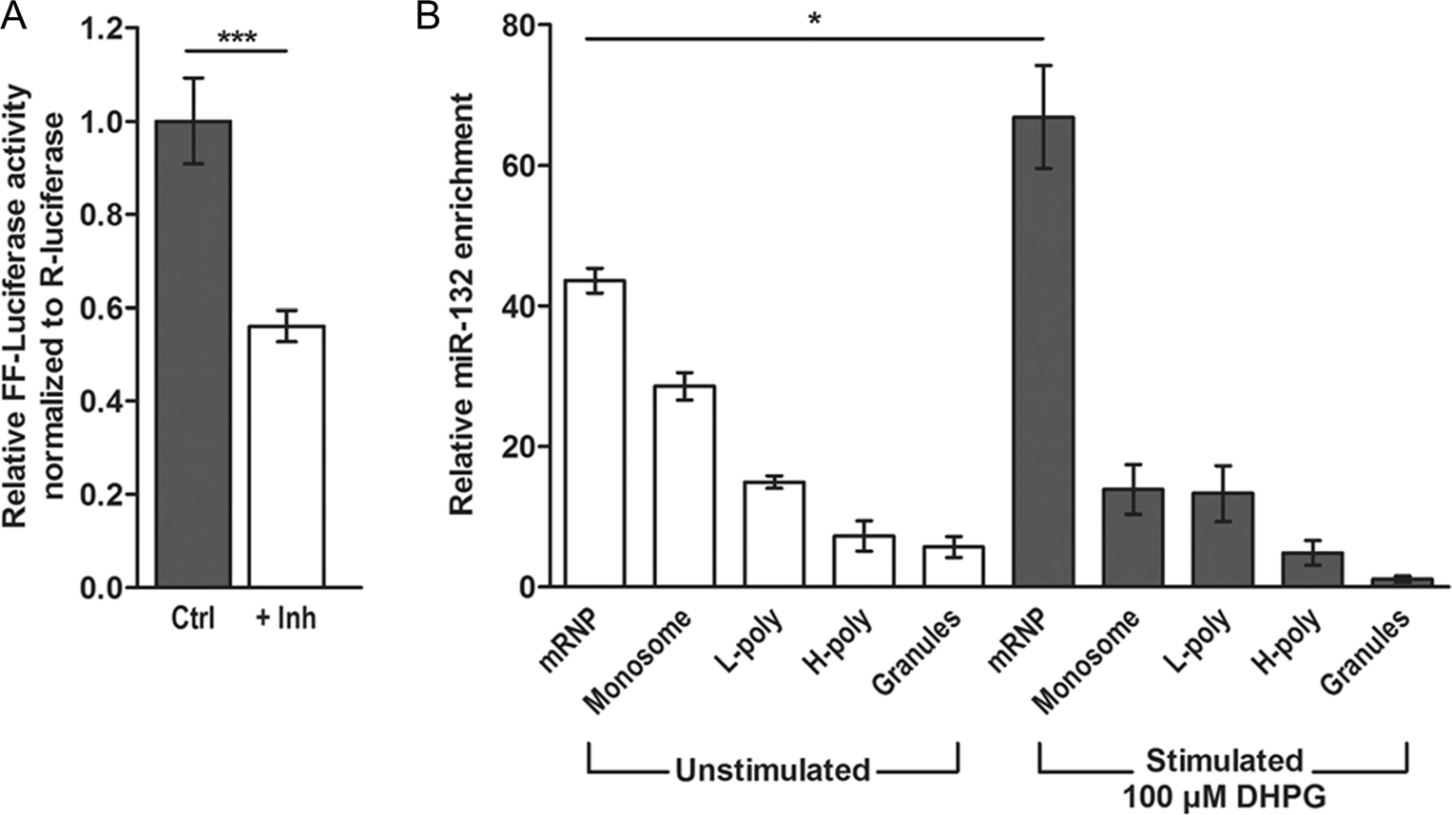
Reduction of the synaptic activity inhibits translation of MMP-9 3’UTR luciferase reporter. **a** Luciferase gene activity in a reporter vector containing the wild-type 3′ UTR of MMP-9 mRNA downstream of a luciferase gene in the control conditions and after the treatment with inhibitors. *Error bars* indicate SEM, *n* = 3, ***p < 0.001 by Student’s *t* test. **b** The level of miR-132 on polisomal fractions isolated from synaptoneurosomes in control conditions and after gp1 mGluR stimulation with DHPG. *Error bars* indicate SEM, *n* = 5, *p < 0.001 by Mann-Whitney test

Primary neurons in culture express spontaneous action potentials that activate the neuronal network and this can be responsible for the baseline expression level of miR-132. Therefore, we have checked whether the diminished neuronal activity will influence the level of endogenous miR-132. We transfected luciferase reporter construct containing the 3’UTR of MMP-9 mRNA and silenced the endogenous neuronal activity, by incubating the cells with a cocktail of inhibitors TTX (1 μM), CNQX (40 μM), APV (100 μM), and nifedipine (5 μM) for 10 h. The reporter responded to the silencing of neuronal activity with the significant decrease of luciferase (Fig. 3a). This result suggests that the association of miR-132 with the 3’UTR of MMP-9 can be regulated by neuronal activity.

In the previous studies, we have shown that MMP-9 mRNA can associate with polyribosomal fractions isolated from stimulated synaptoneurosomes. Here, we investigated the polysomal distribution of endogenous miR-132 from control and DHPG-stimulated synaptoneurosomes. Synaptoneurosomes were incubated with 50 μM DHPG for 20 min, and the polyribosomes were separated by ultracentrifugation on linear sucrose gradient (Fig. 3b). Collected fractions were divided into five groups, which correspond to, from the top to the bottom of the gradient, free messenger ribonucleoprotein (mRNP) complexes; monosomal fraction; light polysomes; heavy polysomes, corresponding to the actively translating polyribosomal fraction; and RNA granules, the heavy fraction that sediments below the polysomes and contains RNA granules [35]. Equal amount of synthetic RNA (U6 snRNA-associated Sm-like protein—LSM from *A. thaliana*) was added to each fraction before RNA extraction for normalization. Total RNA was extracted from the gradient fractions and then analyzed by quantitative PCR. DHPG treatment resulted in the significant increase of miR-132 level in fraction 1, representing mRNPs, what can suggest its dissociation from MMP-9 mRNA which can be now translated and was shown to associate with polyribosomes in stimulated synaptoneurosomes.

### Overexpression of miR-132 in the Mature Neurons Regulates Dendritic Spine Structure by Direct Targeting of MMP-9 mRNA

**Fig. 4:**
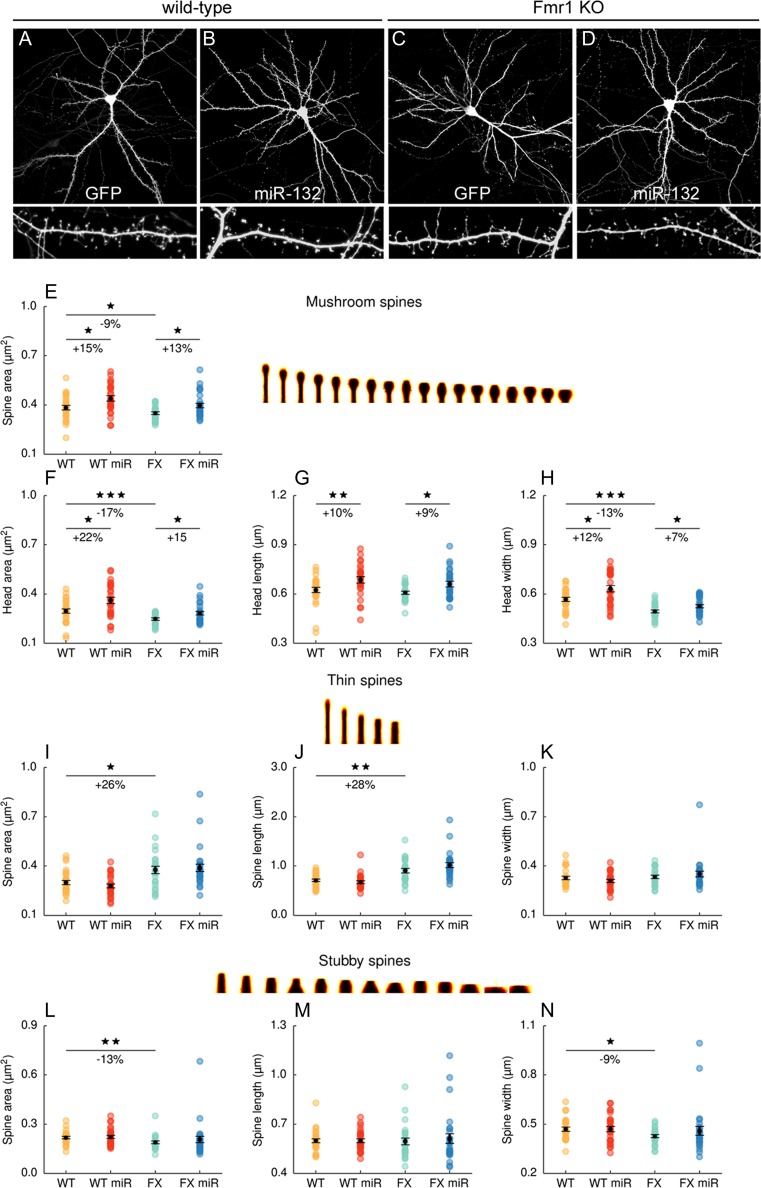
The effect of mir-132 overexpression on the dendritic spines morphology of wt and Fmr1 KO neurons. **a**–**d** Hippocampal neurons isolated from wild-type or Fmr1 KO mice were transfected (DIV14 + 3) with EGFP- or miRNA-132 expressing vectors. Neurons were immunostained with anti-GFP antibody and imaged in the confocal microscope. **e**–**n** Dendritic spine morphology was analyzed, measures such as the spine area, head area, head length, and head width were quantified using the semi-automated spineMagic software for dendritic protrusions clustered into three categories—mushroom spines (**e**–**h**), thin spines (**i**–**k**), and stubby spines (**l**–**n**); 6550 spines were analyzed, *n* = 27 for each experimental condition. *Error bars* indicate SEM, *p < 0.05; ***p < 0.01; ***p < 0.001 by Mann-Whitney test

**Fig. 5:**
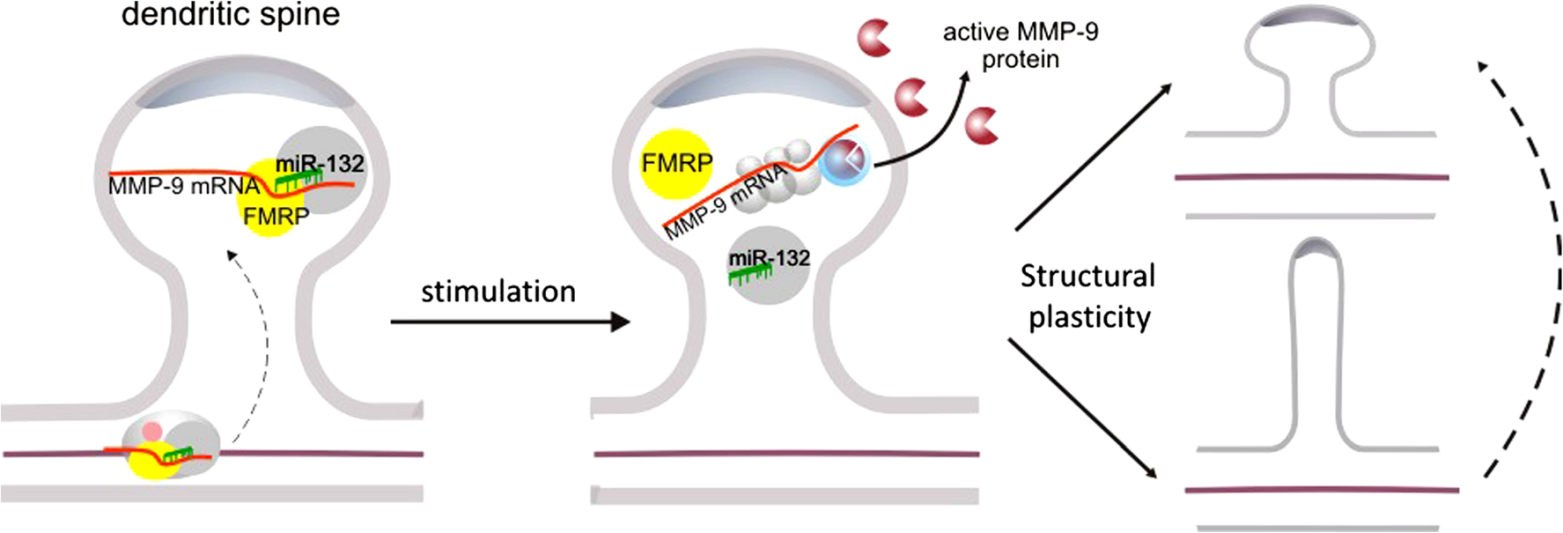
Model depicting the role of miR-132 in the regulation of MMP-9 mRNA translation in response to synaptic stimulation at dendritic spines. FMRP is found in protein-RNA complex with MMP-9 mRNA and miR-132. Based on the published studies [45, 18] and our own results, we hypothesize that activity-dependent release of MMP-9 protein at the synapse is regulated by miR-132 and FMRP, and leads to the structural changes of dendritic spines

The increased level of MMP-9 secreted on dendritic spines has a pronounced effect on synaptic physiology and provoked changes in dendritic spine morphology (elongation of spines). The opposite dendritic spine shape changes were observed due to the miR-132 overexpression [18]. We have previously shown that in the mouse model of fragile X syndrome—Fmr1 KO mice—there is an increased synaptic translation of MMP-9, which results in the higher activity of the enzyme on synapses and contributes to dendritic spine dysmorphologies [22]. Therefore, we used this experimental model of endogenous MMP-9 overexpression to observe the effect of miR-132 on dendritic spine morphology of mature neurons in culture. Hippocampal neurons from wt or Fmr1 KO mice were transfected at 14 days in vitro (DIV14) with plasmid for miR-132 overexpression or plasmid expressing EGFP. Three days after the transfection, neurons were fixed and dendritic spines were visualized and morphologically assessed (Fig. 4a–d). The spines were first sorted into three categories depending on their shape: these which displayed clear heads—mushroom spines, filopodia-like (thin), and small spines without heads (stubby). The parameters measured for long and stubby spines were total spine area, spine length, and spine width. For the mushroom spines, we analyzed spine head parameters—length, width ,and area as well as total spine area. Overexpression of miR-132 in wt and Fmr1 KO neurons resulted in spine head enlargement, when compared to the EGFP transfected controls (Fig. 4e–h). We have observed a significant increase in spine head area (22 % for wt and 15 % for Fmr1 KO), spine head length (10 % for wt and 9 % for Fmr1 KO), and spine head width (12 % for wt and 7 % for Fmr1 KO) after overexpression of miR-132 in wt and Fmr1 KO neurons only for the spines classified as mushroom spines. The shape of thin and stubby spines was not affected by the overexpression of miR-132 (Figs. 4i–n and 5). Significant change in spine shape was also observed between the genotypes—Fmr1 KO neurons had longer filopodia (medium length was increased for about 28 %), the stubby spines were thinner (for about 9 %), and for the mushroom spines the spine head width was reduced (for about 13 %) (*n* = 26; total 6558 spines counted). This data suggest that overexpression of miR-132 in mature hippocampal neurons can regulate structural plasticity of dendritic spines through downregulation of matrix metalloproteinase 9 and may reverse the aberrant morphology of Fmr1 KO spines. We did not observe the significant changes in spine density between wt neurons and Fmr1 KO neurons transfected either with EGFP expressing vector or the one overexpressing miR-132. The average spine densities (spines/10 μm stretch of dendrite) were wt, 4 (±0.9, *n* = 27); wt + miR-132, 4.7 (±1.2, *n* = 27); FX, 4.9 (±1.4, *n* = 27); and FX + miR-132, 4.3 (±1.1, *n* = 27).

## Discussion

In the present study, we provide evidence for the regulation of MMP-9 mRNA by miR-132 in neurons. Our results demonstrate a direct link between the two—not studied together before—important regulators of the synaptic plasticity that underlies learning and memory as well as major neuropsychiatric disorders.

Both miR-132 and MMP-9 are well-described regulators of synaptic plasticity. MMP-9 mRNA undergoes local synthesis at the synapse in response to neuronal stimulation [21] and is released to the extracellular space to regulate structural and functional spine remodeling. Direct influence of recombinant MMP-9 protein on dynamic spine changes has recently been reported [25, 36, 24]. For example, treatment of hippocampal neurons with autoactivating MMP-9 caused elongation and thinning of dendritic spines [24, 25], and the enlargement of dendritic spines in response to chemical long-term potentiation (cLTP) was blocked by MMP-9 inhibitors [37]. Also, the study of Bilousova et al. have demonstrated that a MMP-9 inhibitor—minocycline—promotes maturation of wt and Fmr1 KO dendritic spines both in cultures and in vivo. The beneficial effects of minocycline on dendritic spine morphology were also accompanied by changes in the behavioral performance of Fmr1 KO mice [25]. Finally, in the double Fmr1/MMP-9 knockout mice, the immature phenotype of Fmr1 KO dendritic spines was rescued by genetic elimination of MMP-9 activity [28]. Moreover, the reduction of exaggerated MMP-9 mRNA translation in Fmr1(−/y) mice by the inhibition of translation initiation (reduction of eIF4E phosphorylation) also rescued the dendritic spine morphology defects [29]. The increased MMP-9 activity is observed in neurons as early as 10 min after the stimulation. Synaptic MMP-9 translation may allow for fast regulatory effect on spine morphology and receptor signaling. Our data strongly support a role for miR-132 in the regulation of activity-dependent synaptic translation of MMP-9. In the previous studies, miR-132 has been shown to bind and regulate the MMP-9 3’UTR during the development of the mammary glands in mice [38].

miR-132 was shown to regulate the expression of synaptic mRNAs involved in the modulation of synaptic excitability. Impey and collaborators showed that miR-132-mediated inhibition of a Rho GTPase activator, p250GAP, activates the RAC1-PAK actin-remodeling pathway and thereby regulate activity-dependent spine plasticity in hippocampal neurons [39, 12]. miR-132 can also inhibit the translation of methyl CpG-binding protein 2 (MeCP2) that in turn regulates BDNF expression. Downregulation of MeCP2 level during the post-natal period delays neuronal maturation and synapses formation [40], and opposite—its overexpression triggers dendrite and axon arborization [41, 42]. Another described target of miR-132 is the deacetylase sirtuin 1 (SIRT1) [43]. SIRT1 was recently shown to enhance synaptic plasticity in a mouse hippocampus through a mechanism involving repression of miR-134, resulting in increased expression of BDNF and CREB [44]. The unique feature of MMP-9, the new miR-132 target that we describe here, is that it is secreted at the synapse to directly regulate the morphology of dendritic spines. Our discovery strengthens the importance of the activity-regulated miR-132 as a crucial controller of neuronal plasticity.

In neurons, the role of synaptic microRNAs is believed to be related to the repression of mRNA translation that can be alleviated due to the neuronal stimulation [45]. We observed that translational inhibition of MMP-9 mRNA by miR-132 was regulated by neuronal activity. The expression of luciferase from the reporter vector containing MMP-9 3’UTR was suppressed by the silencing of neuronal network activity of cultured neurons. A similar mechanism was observed for PSD-95 mRNA regulation by miR-125a where the PSD-95 mRNA translation was relieved by activation of gp1 mGluR signaling and dissociation of miR-125a [46]. MicroRNAs have also been shown to copurify with polysomes, what is probably related to their involvement in the repression of translation [47]. In our previous studies, we showed that MMP-9 mRNA is associated with actively translating polyribosomes in synaptoneurosomes and the in vitro stimulation of gp1 mGluR promotes its polysome assembly [22]. Herein, we show that a fraction of miR-132 is associated with polysomes in unstimulated synaptoneurosomes and the stimulation of gp1 mGluRs promotes the association of miR-132 with fraction 1 corresponding to mRNPs. These results suggest that miR-132 can reversibly regulate MMP-9 mRNA translation at synapses downstream of mGluR signaling. Many microRNAs were shown to co purify with polyribosomes in mammalian neurons [48]. In neurons, the local synthesis of synaptic proteins is induced by increased synaptic transmission. Therefore, it is possible that synaptic stimulation leads to the change in the polysome occupancy by specific microRNA and derepression of their target mRNAs. In the mouse model of mature microRNAs, depletion in neurons, the inactivation of the *Dicer1* gene responsible for microRNA maturation, leads to the progressive loss of a whole set of brain-specific miRNAs. Interestingly, the MMP-9 enzymatic activity was upregulated in the brain of mice with disruption of the *Dicer1* gene in forebrain neurons (*Dicer1*^*CaMKCreERT2*^) [49]. This result further supports our hypothesis that microRNAs are responsible for MMP-9 mRNA translational repression.

Regulation of synaptic MMP-9 mRNA translation is crucial for its activity-dependent secretion that enables plastic changes of dendritic spine morphology. Previous studies have shown the effect of miR-132 on dendritic spine shape [18, 6, 11]. Also MMP-9 was shown to regulate spine morphology in wt and Fmr1 KO mice [23–25].

In our study, the overexpression of miR-132 in neurons led to the increase in the dendritic spines heads; however, modulation of spine shape was more pronounced in case of wild-type cells than Fmr1 KO neurons. Interestingly, the width of the spine head increased for about 12 % and the spine head area for 22 % for wild-type dendritic spines, while for the Fmr1 KO neurons it was 7 and 15 % respectively. This would implicate that the FMRP has an additional regulatory effect on the miR-132-MMP-9 interaction. An example of such regulatory mechanism is PSD-95 mRNA shown to be regulated by miR-125b and FMRP activity-dependent phosphorylation [45]. Further study is needed to reveal this question for MMP-9 mRNA and miR-132.

In aggregate, in the present study, we provide evidence that neuronal stimulation-driven rapid MMP-9 protein synthesis can be an effect of miR-132-dependent derepression of MMP-9 mRNA. Our findings provide an insight into a molecular mechanism that involves miR-132-regulated MMP-9 expression in neurons and their function for structural plasticity of dendritic spines.
